# Author Correction: Automatic diagnosis of the 12-lead ECG using a deep neural network

**DOI:** 10.1038/s41467-020-16172-1

**Published:** 2020-05-01

**Authors:** Antônio H. Ribeiro, Manoel Horta Ribeiro, Gabriela M. M. Paixão, Derick M. Oliveira, Paulo R. Gomes, Jéssica A. Canazart, Milton P. S. Ferreira, Carl R. Andersson, Peter W. Macfarlane, Wagner Meira, Thomas B. Schön, Antonio Luiz P. Ribeiro

**Affiliations:** 1 Universidade Federal de Minas Gerais, Belo Horizonte, Brazil; 2 Uppsala University, Uppsala, Sweden; 3 Telehealth Center from Hospital das Clínicas da Universidade Federal de Minas Gerais, Belo Horizonte, Brazil; 4 University of Glasgow, Glasgow, Scotland

**Keywords:** Cardiology, Electrodiagnosis, Computational science

Correction to: *Nature Communications* 10.1038/s41467-020-15432-4, published online 9 April 2020.

The original version of this Article contained an error in the spelling of the author Wagner Meira Jr., which was incorrectly given as Meira Wagner Jr. This has now been corrected in both the PDF and HTML versions of the Article.

