# Supplementary material for: Automatic diagnosis of the 12-lead ECG using a deep neural network
Source: Nat Commun. 2020 Apr 9;11:1760. doi: 10.1038/s41467-020-15432-4 (PMC7145824; doi:10.1038/s41467-020-15432-4)
Supplement: Supplementary file 1 — Supplementary Information [file 41467_2020_15432_MOESM1_ESM.pdf]

Supplementary Information: Automatic diagnosis of the 12-lead ECG using a deep neural network

Antônio H. Ribeiro[a, b, 1], **Manoel Horta Ribeiro**[a], **Gabriela M.M. Paixão**[a, c], **Derick M. Oliveira**[a], **Paulo R. Gomes**[c], **Jéssica A. Canazart**[a, c], **Milton P. S. Ferreira**[a, c], **Carl R. Andersson**[b], **Peter W. Macfarlane**[d], **Wagner Meira Jr.**[a], **Thomas B. Schön**[b], and **Antonio Luiz P. Ribeiro**[a, c, 2]

[a]Universidade Federal de Minas Gerais, Brazil; [b]Uppsala University, Sweden; [c]Telehealth Center from Hospital das Clínicas da Universidade Federal de Minas Gerais, Brazil; [d]Glasgow University, Scotland; [1]antonio-ribeiro@ufmg.br; [2]tom@hc.ufmg.br

		predicted label							
		DNN		cardio.		emerg.		stud.	
	true label	not present	present	not present	present	not present	present	not present	present
1dAVb	not present	795	4	797	2	786	13	782	17
	present	2	26	9	19	5	23	2	26
RBBB	not present	789	4	788	5	792	1	790	3
	present	0	34	1	33	8	26	2	32
LBBB	not present	797	0	797	0	796	1	795	2
	present	0	30	3	27	4	26	3	27
SB	not present	808	3	808	3	808	3	807	4
	present	1	15	1	15	2	14	4	12
AF	not present	814	0	811	3	812	2	805	9
	present	3	10	3	10	5	8	1	12
ST	not present	788	2	789	1	788	2	787	3
	present	1	36	7	30	2	35	6	31

Supplementary Table 1. (Confusion matrices) Show the absolute number of: i) false posives; ii) false negatives; iii) true positives; and, iv) true negatives, for each abnormality on the test set.

	1dAVb	RBBB	LBBB	SB	AF	ST
DNN vs cardio.	0.656	0.917	0.945	0.830	0.780	0.864
DNN vs emerg.	0.684	0.792	0.909	0.796	0.595	0.930
DNN vs stud.	0.642	0.928	0.912	0.760	0.574	0.855
cardio. vs emerg.	0.656	0.824	0.923	0.912	0.515	0.847
cardio. vs stud.	0.612	0.871	0.889	0.880	0.700	0.792
emerg. vs stud.	0.615	0.799	0.852	0.907	0.508	0.897

(a)

	1dAVb	RBBB	LBBB	SB	AF	ST
DNN vs Cert. cardiol. 1	0.758	0.928	0.964	0.770	0.696	0.847
DNN vs Certif. cardiol. 2	0.852	0.942	1.000	0.770	0.746	0.884
Cert. cardiol. 1 vs Certif. cardiol. 2	0.741	0.955	0.964	0.844	0.831	0.902

(b)

Supplementary Table 2. (Kappa coefficients) Show the Kappa scores measuring the inter-rater agreement on the test set. In (a), we compare the DNN, the medical residents and the students two at a time. In (b), we compare the DNN, and the certified cardiologists that annotated the test set (`certif. cardiol.`). If the raters are in complete agreement then it is equal to 1. If there is no agreement among the raters other than what would be expected by chance it is equal to 0.

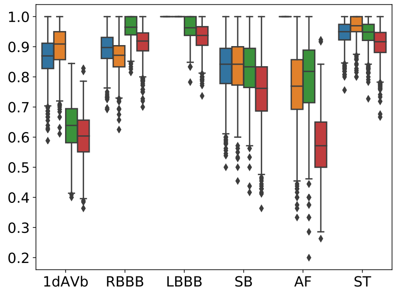

(a) Precision (PPV)

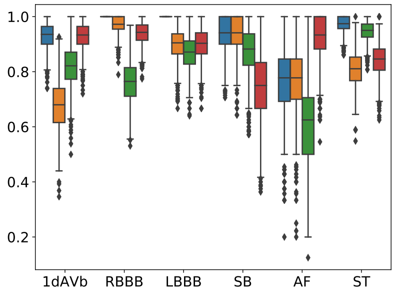

(b) Recall (Sensitivity)

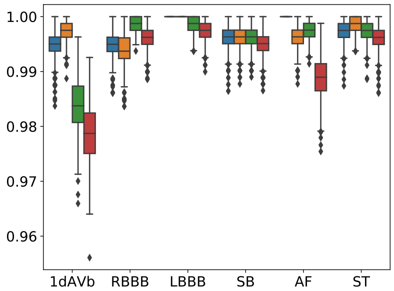

(c) Specificity

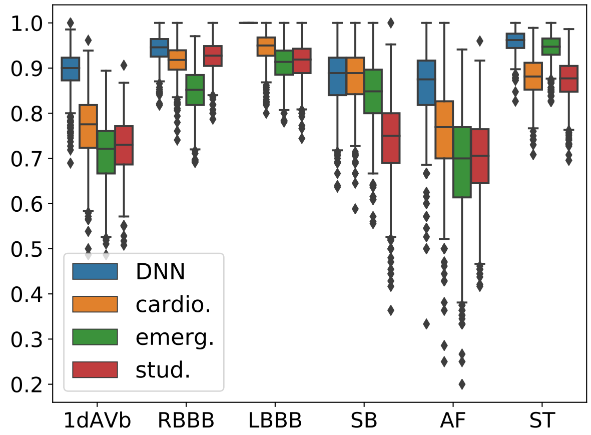

(d) F1 score

Supplementary Figure 1. (Bootstrapped scores) Display boxplots of empirical distribution of precision, recall, specificity and F1 score on the test set. Sampling with replacement (i.e. bootstrapping) from the test set was used to generate $n = 1000$ samples. The results are given for the DNN, the medical residents and students. Source data are provided as a Source Data file. The boxplots should be read as follows: the central line correspond to the median value of the empirical distribution, the box region correspond to the range of values between the first and third quartile (also knonw as interquartile range or IQR), the whiskers extend from 1.5 IQR below and above the firsth and third quartiles, values outside of that range are considered outliers and show as diamonds.

	1dAVb	RBBB	LBBB	SB	AF	ST
DNN vs cardio.	0.225	0.414	0.083	1.000	0.180	0.096
DNN vs emerg.	**0.007**	0.166	0.025	0.705	0.157	0.655
DNN vs stud.	**0.009**	0.655	0.025	0.157	0.052	0.058
cardio. vs emerg.	0.108	0.366	0.317	0.564	0.763	0.206
cardio. vs stud.	0.102	0.739	0.414	**0.046**	0.206	0.782
emerg. vs stud.	0.853	0.248	1.000	0.083	0.439	0.059

Supplementary Table 3. (McNemar test) Display the *p*-values for the McNemar test comparing the misclassification on the test set. The DNN, the medical residents and the students were compared two at a time. Entries with statistical significance (with 0.05 significance level) are displayed in **boldface**.

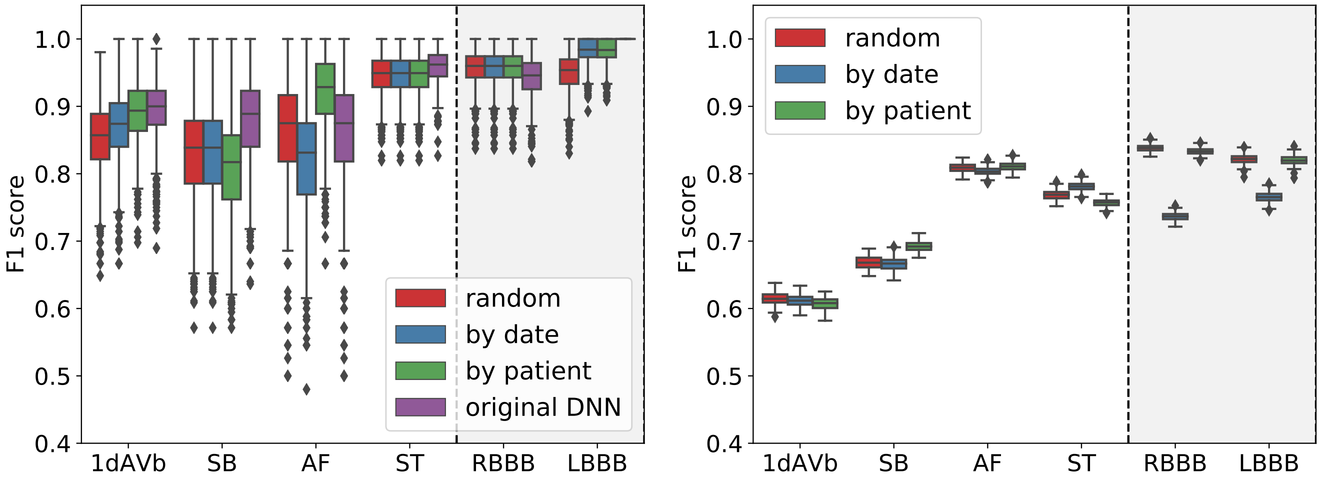

(a) original test set **(b)** secondary test set (last 5% split)

Supplementary Figure 2. (Bootstrapped scores for alternative splits) Boxplots for the bootstrapped F_1 scores for the DNN using alternative 90%-5%-5% splits for training, validation and as a *secondary* test set. For the splits ordered: randomly; by date; and, stratified by patients. In all cases, the performance is evaluated on: (a) the original test set for $n = 1000$ bootstraped samples; and, on (b) the secondary test set (last 5% split) for $n = 200$ bootstraped samples. On (a), we also present the original DNN performance for comparison, which was developed using a 98%-2% split. The performance gap between (a) and (b) is due to the difference in the gold standard. The secondary test set obtained from the last 5% has a less accurate annotation, since it has not been annotated by multiple doctors and it uses natural language processing to extract the diagnosis from a written report. This extra noise result in worse F_1 score in (b) when compared with (a). On the other hand, the secondary 5% test split contain more than 100,000 records, which yield more stable performance in the bootstrap analysis, with more concentrated empirical distributions for the F_1 score. Both RBBB and LBBB (highlighted on the plot) present on (b) a statistically significant difference between the performance of the split ordered by date and the other two splits, that difference is due to some changes in personal that took place in the the Telehealth center, that affected the period used in the test set (10-2016 to 06-2017), resulting in lower annotation quality. A certified cardiologist reviewed cases for which the neural network have been considered wrong when compared to the gold standard from the 5% split collected from 10-2016 to 06-2017, 100 supposedly wrong RBBB and 100 supposedly wrong LBBB. The certified cardiologist reported that the neural network is actually correct, respectively, 86% and 83% percent of the cases. This analysis show the importance of a test set with a good annotation quality to obtain reliable estimation of the DNN performance. And, also, that periods of lower annotation quality in the dataset are overcome by a very high number of examples. Source data are provided as a Source Data file. See Supplementary Figure 1 caption for the definition of all elements in the boxplot.

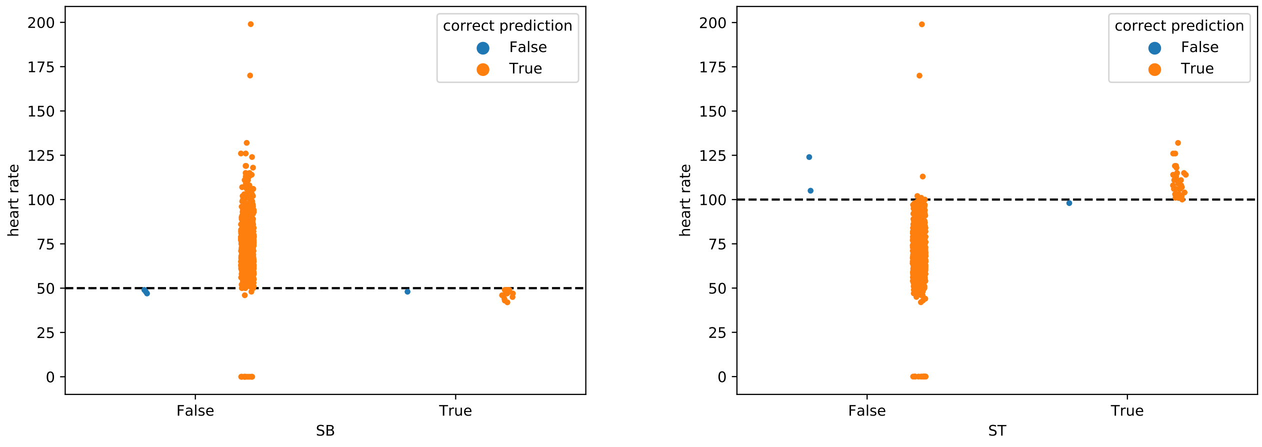

(a) **(b)**

Supplementary Figure 3. (Heart rate *vs* DNN predictions) Heart rate measured by the Uni-G software for samples in the test set is given on the y-axis. The color indicates if the *DNN* make the correct prediction or not. The x-axis separates the dataset accordingly to the presence of: SB in (a); and, ST in (b). A horizontal line show the threshold of 50 bpm for SB in (a); and, of 100 bpm for ST in (b), which delimit the consensus definition of SB and ST. Notice that most exams for which the neural network fails to get the correct result are very close to this threshold line and are the borderline cases we mentioned in the discussion. It should be highlighted that this automatic measurement system is not perfect, and measurements that may indicate some of the conditions do not necessarily agree with our board of cardiologist (e.g. there are exams with heart rate above 100 acording to Uni-G that are not classified by our cardiologist as ST).
