# Supplementary material for: Automatic diagnosis of the 12-lead ECG using a deep neural network
Source: Nat Commun. 2020 Apr 9;11:1760. doi: 10.1038/s41467-020-15432-4 (PMC7145824; doi:10.1038/s41467-020-15432-4)
Supplement: Supplementary file 3 — Reporting Summary [file 41467_2020_15432_MOESM3_ESM.pdf]

nature research

Corresponding author(s): Antonio Luiz Ribeiro

Last updated by author(s): 2020-01-20

Reporting Summary

Nature Research wishes to improve the reproducibility of the work that we publish. This form provides structure for consistency and transparency in reporting. For further information on Nature Research policies, see Authors & Referees and the Editorial Policy Checklist.

Statistics

For all statistical analyses, confirm that the following items are present in the figure legend, table legend, main text, or Methods section.

n/a	Confirmed	
☐	☒	The exact sample size (*n*) for each experimental group/condition, given as a discrete number and unit of measurement
☐	☒	A statement on whether measurements were taken from distinct samples or whether the same sample was measured repeatedly
☐	☒	The statistical test(s) used AND whether they are one- or two-sided<br>*Only common tests should be described solely by name; describe more complex techniques in the Methods section.*
☒	☐	A description of all covariates tested
☒	☐	A description of any assumptions or corrections, such as tests of normality and adjustment for multiple comparisons
☒	☐	A full description of the statistical parameters including central tendency (e.g. means) or other basic estimates (e.g. regression coefficient) AND variation (e.g. standard deviation) or associated estimates of uncertainty (e.g. confidence intervals)
☒	☐	For null hypothesis testing, the test statistic (e.g. *F*, *t*, *r*) with confidence intervals, effect sizes, degrees of freedom and *P* value noted<br>*Give P values as exact values whenever suitable.*
☒	☐	For Bayesian analysis, information on the choice of priors and Markov chain Monte Carlo settings
☒	☐	For hierarchical and complex designs, identification of the appropriate level for tests and full reporting of outcomes
☒	☐	Estimates of effect sizes (e.g. Cohen's *d*, Pearson's *r*), indicating how they were calculated

Our web collection on statistics for biologists contains articles on many of the points above.

Software and code

Policy information about availability of computer code

Data collection	Telehealth network infrastructure and software was used for collecting and storing the data. This software was developed in-house and it is not the object of this study.
Data analysis	Custom software was used for for training, validating and testing our model. This custom software is openly available and was built on Python using standard, open-source, scientific libraries. Check code availability statement.

For manuscripts utilizing custom algorithms or software that are central to the research but not yet described in published literature, software must be made available to editors/reviewers. We strongly encourage code deposition in a community repository (e.g. GitHub). See the Nature Research guidelines for submitting code & software for further information.

Data

Policy information about availability of data

All manuscripts must include a data availability statement. This statement should provide the following information, where applicable:

- Accession codes, unique identifiers, or web links for publicly available datasets
- A list of figures that have associated raw data
- A description of any restrictions on data availability

Upon publication, the test data used to support the findings of this study will be made publicly available without restrictions. The DNN model parameters that give the results presented in this paper will also be made available without restrictions. Restrictions apply to the availability of the training set, for which requests to access the training data will be considered on an individual basis by the Telehealth Network of Minas Gerais. Any data use will be restricted to non-commercial research purposes, and the data will only be made available on execution of appropriate data use agreements.

Field-specific reporting

Please select the one below that is the best fit for your research. If you are not sure, read the appropriate sections before making your selection.

☒ Life sciences ☐ Behavioural & social sciences ☐ Ecological, evolutionary & environmental sciences

For a reference copy of the document with all sections, see nature.com/documents/nr-reporting-summary-flat.pdf

Life sciences study design

All studies must disclose on these points even when the disclosure is negative.

Sample size	The sample of this is study is a large dataset consisting of 2,322,513 ECG records from 1,676,384 different patients of 811 counties in the state of Minas Gerais/Brazil, from the Telehealth Network of Minas Gerais. A sample of 827 exams were using for testing of the DNN performance and compare it with medical residents and students. Considering the large sample used, no sample size calculation was performed.
Data exclusions	Data from patients of 16 years-old or less were excluded, considering that the reference values and criteria for diagnosis in children and adolescents are different from those used in adults.
Replication	The study consists of training a Deep Neural Network, to validate in a subsample and to test in a independent sample. No further replication of the study was done.
Randomization	Randomization does not apply to the present study.
Blinding	Blinding does not apply to the present study.

Reporting for specific materials, systems and methods

We require information from authors about some types of materials, experimental systems and methods used in many studies. Here, indicate whether each material, system or method listed is relevant to your study. If you are not sure if a list item applies to your research, read the appropriate section before selecting a response.

Materials & experimental systems

n/a	Involved in the study	
☒	☐	Antibodies
☒	☐	Eukaryotic cell lines
☒	☐	Palaeontology
☒	☐	Animals and other organisms
☐	☒	Human research participants
☒	☐	Clinical data

Methods

n/a	Involved in the study	
☒	☐	ChIP-seq
☒	☐	Flow cytometry
☒	☐	MRI-based neuroimaging

Human research participants

Policy information about studies involving human research participants

Population characteristics	The study population the provided the ECGs for this study is composed by user of the public health system in the state of Minas Gerais. The state of Minas Gerais had, in the census of 2010, a population of 19,597,330 persons; our sample is composed of 1,676,384 different patients of 811 counties of the state that used the tele-cardiology service of the Telehealth Network of Minas Gerais. The general feaures of this sample is described in Table 1, include age and sex distribution.
Recruitment	Most of the remote points of the Tlehealth network of Minas Gerais are located in primary health care centers, although some ECGs machines are located in emergency units and ambulances. Data of the clinical assistance kept by the Telehealth Center was used in this study. No specific recruitment procedure was used.
Ethics oversight	This study was approved by the Research Ethics Committee of the Universidade Federal de Minas Gerais, protocol 49368496317.7.0000.5149. Since this is a study with secondary data, no informed consent was required.

Note that full information on the approval of the study protocol must also be provided in the manuscript.
